# Quantitative Assessment of Intervertebral Disc Composition by MRI: Sensitivity to Diurnal Variation

**DOI:** 10.3390/tomography9030084

**Published:** 2023-05-16

**Authors:** Hiroyuki Hamaguchi, Maho Kitagawa, Daiki Sakamoto, Ulrich Katscher, Hideki Sudo, Katsuhisa Yamada, Kohsuke Kudo, Khin Khin Tha

**Affiliations:** 1 Laboratory for Biomarker Imaging Science, Graduate School of Biomedical Science and Engineering, Hokkaido University, N15 W7, Kita-ku, Sapporo 060-8638, Japan; 2 Philips Research Laboratories, Roentgenstrasse 24-26, 22335 Hamburg, Germany; 3 Department of Orthopaedic Surgery, Hokkaido University Hospital, N14 W5, Kita-ku, Sapporo 060-8648, Japan; 4 Department of Diagnostic Imaging, Hokkaido University Hospital, N14 W5, Kita-ku, Sapporo 060-8648, Japan; 5 Global Center for Biomedical Science and Engineering, Faculty of Medicine, Hokkaido University, N15 W7, Kita-ku, Sapporo 060-8638, Japan

**Keywords:** intervertebral disc, lumbar, T1ρ, apparent diffusion coefficient, electrical conductivity, MRI, electric properties tomography, diffusion-weighted imaging, diurnal, quantitative

## Abstract

Whether diurnal variation exists in quantitative MRI indices such as the T1rho relaxation time (T1ρ) of the intervertebral disc (IVD) is yet to be explored. This prospective study aimed to evaluate the diurnal variation in T1ρ, apparent diffusion coefficient (ADC), and electrical conductivity (σ) of lumbar IVD and its relationship with other MRI or clinical indices. Lumbar spine MRI, including T1ρ imaging, diffusion-weighted imaging (DWI), and electric properties tomography (EPT), was conducted on 17 sedentary workers twice (morning and evening) on the same day. The T1ρ, ADC, and σ of IVD were compared between the time points. Their diurnal variation, if any, was tested for correlation with age, body mass index (BMI), IVD level, Pfirrmann grade, scan interval, and diurnal variation in IVD height index. The results showed a significant decrease in T1ρ and ADC and a significant increase in the σ of IVD in the evening. T1ρ variation had a weak correlation with age and scan interval, and ADC variation with scan interval. Diurnal variation exists for the T1ρ, ADC, and σ of lumbar IVD, which should be accounted for in image interpretation. This variation is thought to be due to diurnal variations in intradiscal water, proteoglycan, and sodium ion concentration.

## 1. Introduction

Intervertebral discs (IVDs) are fibrocartilaginous structures that form articulation between adjacent vertebral bodies and are present from the second cervical vertebra through the first sacral vertebra [1]. Each intervertebral disc (IVD) allows movement between adjacent vertebral bodies, absorbs shock, and transmits loads through the vertebral column [2]. IVDs are developed as complex structures with specific compositions to enable these functions: IVDs are formed from a central gelatinous mass called nucleus pulposus (NP), a fibrous outer ring called annulus fibrosus (AF), and a cartilaginous vertebral end plate covering the superior and inferior surfaces [1,2]. Water, proteoglycans, and collagen are the main constituents of IVD. Their depletion is reported in aging and degenerative disc diseases [2,3].

Magnetic resonance imaging (MRI) is widely used to evaluate the integrity of IVD noninvasively. Pfirrmann grade, visually evaluated using T2-weighted images, has long been considered a standard quantifier [4]. However, visual assessment demands expertise and is not always free of observer bias. Moreover, prior studies have reported the lack of correlation of Pfirrmann grade with clinical symptoms such as low back pain [5,6]. Therefore, evaluation using unbiased quantitative indices is desired. Complementary to this, recent MRI studies have shown the potential usefulness of quantitative MRI techniques such as T1rho (T1ρ) imaging and diffusion-weighted imaging (DWI) for the evaluation of IVD integrity. T1ρ value, derived from T1ρ imaging, is known to be highly sensitive to the changes in intradiscal proteoglycan concentration, and the apparent diffusion coefficient (ADC) derived from DWI is sensitive to the changes in water content [7,8]. A few initial studies have also suggested the potential role of sodium imaging due to the strong affinity of proteoglycans and water to sodium ions [9].

Quantitative assessment by MRI requires normative values [10]. The normative T1ρ and ADC values of lumbar IVDs have been reported [7,8,11], but less attention has been paid to their diurnal variation. In fact, IVD experiences a daily change in intradiscal pressure according to the variation in day and night activity [12]. A decrease in IVD height in the evening, a common observation, has been considered to be due to this difference [13]. The quantitative MRI indices may also exhibit diurnal variation, which can further affect the decision of whether an IVD of interest is normal or abnormal. To our knowledge, there have been only a few studies on the diurnal variation in the ADC values of lumbar IVDs [14,15,16]. It is poorly known if a diurnal variation exists in T1ρ and sodium ion concentration. The relationship among these quantitative MRI indices is also not known. Knowledge of the diurnal variation in these quantitative indices is essential for clinical decisions and understanding the metabolic regulation of major constituents of IVD.

This prospective study aimed to evaluate the diurnal variation in the T1ρ, ADC, and electrical conductivity (σ) of adult lumbar IVD and their relationship with one another and other MRI or clinical indices. σ, derived from electric properties tomography (EPT), was chosen to reflect sodium ion concentration noninvasively. EPT is a recently developed MRI technique that can map the σ of tissues by merely post processing the images of standard MRI sequences [17]. σ is known to be highly sensitive to changes in sodium ion concentration. Measuring sodium ion concentration by sodium imaging requires a dedicated coil, which is not usually available in clinical MR systems [9]. On the other hand, the estimation of σ by EPT does not need a special coil or intervention, thus making it easy to implement in any clinical MR system.

## 2. Materials and Methods

### 2.1. Participants

This prospective study was approved by the institutional review board of Hokkaido University Hospital, Sapporo, Japan (protocol code 017-0455). Written informed consent was obtained from all participants.

Over ten months, sedentary workers engaging in routine nonstrenuous daytime activities were recruited. The exclusion criteria were absolute contraindications to MRI, low back pain that prevented the volunteer from lying still during MRI, and known pathologies of the lumbar IVD. Seventeen subjects (fourteen men and three women) aged 25–48 years (mean age = 33.41 ± 5.70 years) were included in the study. According to an 11-point numeric pain rating scale, the mean low back pain score of the volunteers on the day of the MRI was 1.12 ± 1.32 (range = 0–3) [18]. Their mean body mass index (BMI) was 21.12 ± 2.24.

### 2.2. MRI

All examinations were conducted using a 3T scanner (Achieva TX, Philips Healthcare, Best, The Netherlands) and a 32-channel torso/radiofrequency (RF) coil. The scan range was set to include all lumbar vertebrae and sacrum.

Sagittal 2D fast spin-echo T2-weighted imaging {T2WI; repetition time (TR)/echo time (TE) = 4000/90 ms}, 3D ultrafast gradient echo T1ρ imaging (TR/TE = 5.8/0.944 ms, flip angle (FA) = 15°, spin-lock frequency = 500 Hz, spin-lock time (TSL) = 0, 20, 40, 80 ms, in-plane resolution = 1.56 mm × 1.56 mm, the number of signal averages (NSA) = 1), 2D fast spin-echo echo-planar DWI (TR/TE = 3100/71 ms, b = 0, 1000 s/mm^2^, in-plane resolution = 1.56 mm × 1.54 mm, NSA = 6), and 2D steady-state free precession EPT (TR/TE = 3.04/1.52 ms, FA = 30°, in-plane resolution = 1.92 mm × 1.93 mm, NSA = 40 with the first 10 averages serving as start-up cycles) were acquired twice, once in the morning before engaging in the participants’ regular activities (i.e., between 7:00 a.m. and 8:30 a.m.) and once in the evening after the activities (i.e., between 5:00 p.m. and 8:30 p.m.). The average interval between the two scans was 10.78 ± 0.66 h (range = 9.93–12.23 h). Each scan lasted about 20 min.

In addition to the above scans, T1ρ imaging, DWI, and EPT were repeated on a volunteer in the same scan session to evaluate the repeatability of the measurements.

### 2.3. Image Reconstruction

Images of each sequence (i.e., T1ρ imaging or DWI) were co-registered before the reconstruction of corresponding maps at the operator console or using SPM12 (Wellcome Trust Centre for Neuroimaging, University College of London, Oxford, UK).

T1ρ maps were reconstructed voxel-by-voxel from the T1ρ imaging data, using the formula M_TSL_ = M_0_ × e^−TSL/T1ρ^, where M_TSL_ and M_0_ denoted T1ρ spin lock prepared magnetization for each TSL and the equilibrium magnetization, respectively. Reconstruction was performed at a standalone workstation (Jim8, Xinapse Systems Ltd., Essex, UK).

ADC maps were reconstructed from the DWI data using the formula S_b_ = S_0_ × e^−b.ADC^, where S_b_ = signal intensity of co-registered diffusion-weighted images at the given b-value, S_0_ = signal intensity on echo-planar T2-weighted images without diffusion weighting (i.e., b = 0 s/mm^2^), and b = diffusion weighting. This reconstruction was performed on the main operator console of the MRI scanner.

Using a standalone computer, σ maps were reconstructed from the phase of EPT data, using the formula σ ≅ ∂_x_^2^ φ/(2μ_0_ ω), where φ = transceive phase, μ_0_ = magnetic vacuum permeability, and ω = Larmor frequency (128 MHz for 3T). This formula differs from the general version of EPT, which is based on numerically solving the truncated Helmholtz equation σ = ∇2φ/(2 μ_0_ω) = (∂_x_^2^ + ∂_y_^2^ + ∂_z_^2^)φ/(2 μ_0_ω). The partial derivatives in the three spatial directions x, y, and z require a volumetric magnetic resonance (MR) image so that the approximation σ ≅ (∂_x_^2^ + ∂_y_^2^) φ/(2 μ_0_ω) can be proposed to allow EPT on fast 2D scans with x and y defining the imaging plane [17,19]. The approximation was limited to only along the axis parallel to each IVD (termed x in the aforementioned equation) to avoid the contamination of the reconstructed σ with boundary effects from the derivative perpendicular to the IVD. Semiautomatic segmentation of each IVD (i.e., the IVD between the first and second lumbar vertebrae through that between the fifth lumbar vertebra and sacrum), using the magnitude images of EPT as a reference, was performed before reconstruction. The required second derivative of the transceive phase was performed numerically with a differentiation kernel of 10 voxels along x. The typical noise-enhancing effect of numerical differentiation was counterbalanced with a subsequent bilateral median filter with a kernel size shaped locally to voxels having ±10% of the signal magnitude as the target voxels.

### 2.4. Visual Assessment

MR images of the lumbar region can suffer from various artifacts, including flow and motion artifacts. Thus, all images and maps were visually evaluated by a radiologist (23 years of experience) and radiological technologist (15 years of experience) for any artifacts. Only artifact-free images were used for quantitative analysis. The radiologist, unaware of the mapping results, determined the Pfirrmann grade of each lumbar IVD using mid-sagittal T2WI [4].

### 2.5. Region-of-Interest Setup and Measurements

**Figure 1:**
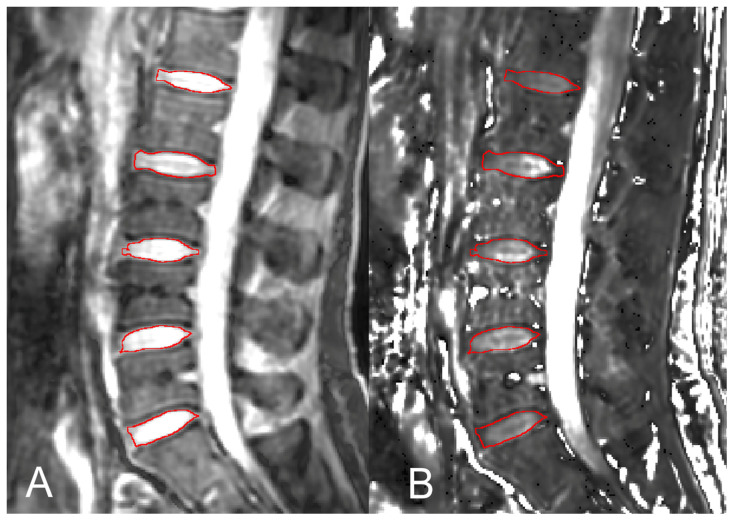
Regions-of-interest (ROIs) setup for T1rho (T1ρ) maps. ROIs (circled in red) were drawn semiautomatically on the images with spin-lock time (TSL) = 0 ms (**A**). The ROIs were then superimposed onto the corresponding T1ρ maps for measurements (**B**).

Regions of interest (ROIs) were placed on each lumbar IVD (i.e., from the IVD between the first and second lumbar vertebrae through that between the fifth lumbar vertebra and the sacrum). ROIs for T1ρ and ADC measurements were placed semiautomatically on the mid-sagittal images of each MRI sequence, using Jim8. ROIs were drawn on T1ρ images at TSL = 0 ms and echo-planar images with no diffusion weighting (b = 0 s/mm^2^), which provided maximum image contrast. Special attention was paid so that each ROI included the entire lumbar IVD while avoiding the inclusion of nearby structures. The ROIs were then superimposed onto the corresponding co-registered maps. The accuracy of the ROI position was confirmed by a radiological technologist (15 years of experience). Example ROIs are given in Figure 1. For σ maps, ROIs were set to include the entire segmented IVDs.

For each sequence, the mean value within each ROI was then obtained. The voxels with ROIs with values exceeding the mean ± 5 standard deviations were considered artifactual or noise and discarded.

The IVD height index was also measured to evaluate the relationship between the diurnal variation in the quantitative MRI indices and that of IVD height. A trained radiological technologist (4 years of experience) performed the measurements on a standalone DICOM viewer (RadiAnt DICOM Viewer, Medixant, Poznan, Poland). The steps followed those detailed in a previous report [20].

### 2.6. Statistical Analysis

Repeatability of T1ρ, ADC, and σ measurements of IVDs was tested in the volunteer in whom two repeated scans were performed in the same scan session. Intraclass correlation coefficient (ICC) was used to determine the repeatability, and ICC greater than 0.75 was considered repeatable [21].

T1ρ, ADC, σ, and IVD height index of all lumbar IVDs were compared between the two time points, using paired t or Wilcoxon signed-rank tests. The relationships of each index between the two time points and the association of diurnal variation (Δ) of each MRI index, given by the difference of their values between the two time points, with Δ of other quantitative MRI indices, Δ of IVD height index, age, Pfirrmann grade, BMI, IVD level, and the interval between the two scans (hereafter scan interval), were tested using Pearson’s product moment correlation analysis. Variation in the degree of Δ among IVD levels was evaluated using repeated measures analysis of variance (ANOVA). For all comparisons, *p* < 0.05 was considered statistically significant. Statistical analysis was conducted using Statistical Package for the Social Sciences (SPSS) software version 26 (IBM, New York, NY, USA).

## 3. Results

Altogether, 85 lumbar IVDs were evaluated. Of these, T1ρ measurement was possible in all IVDs. A total of 84 IVDs were valid for ADC, and 83 IVDs for σ. The Pfirrmann grades of the lumbar IVDs were 7, 70, and 8 for grades II, III, and IV, respectively.

The ICC values for the T1ρ, ADC, and σ measurements were 0.874, 0.858, and 0.974, suggesting good to excellent repeatability.

**Figure 2:**
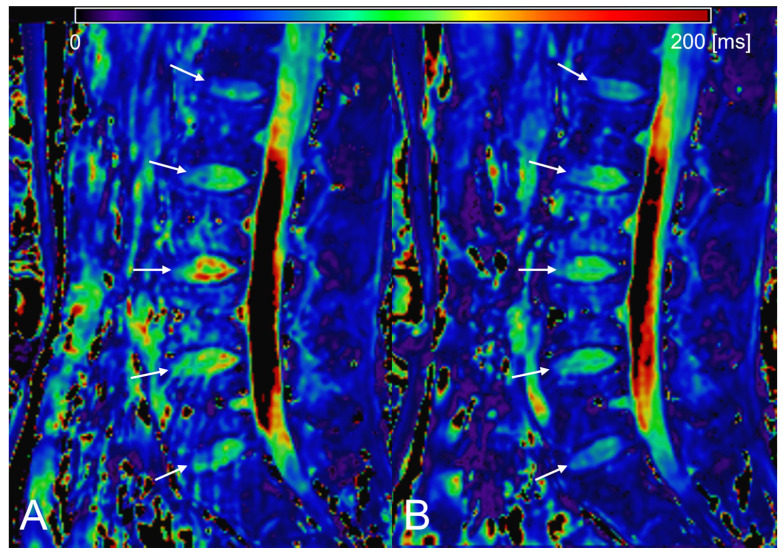
Diurnal variation in T1rho (T1ρ) of lumbar intervertebral discs (IVDs). T1ρ maps of the morning scan (**A**) and evening scan (**B**) are given. The white arrows indicate IVDs. The look-up table represents T1ρ value in ms. T1ρ of the evening scan is lower than the morning scan.

**Figure 3:**
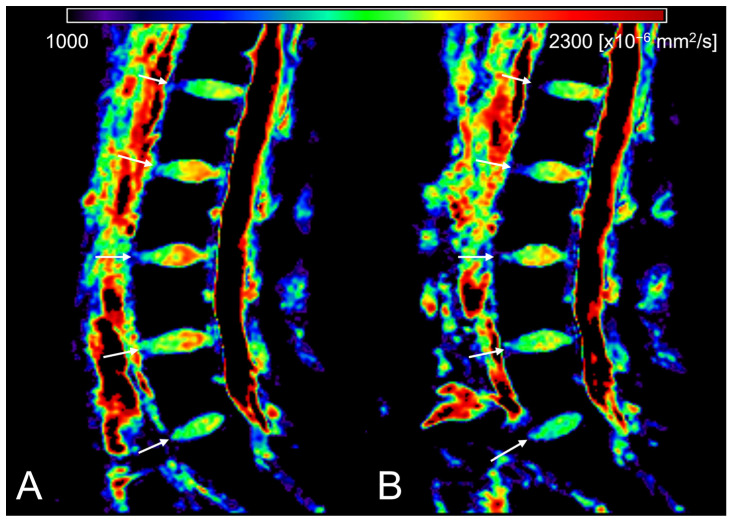
Diurnal variation in apparent diffusion coefficient (ADC) of lumbar intervertebral discs (IVDs). ADC maps of the morning scan (**A**) and evening scan (**B**) are given. The white arrows indicate IVDs. The look-up table represents ADC value in mm^2^/s. ADC of the evening scan is lower than the morning scan.

**Figure 4:**
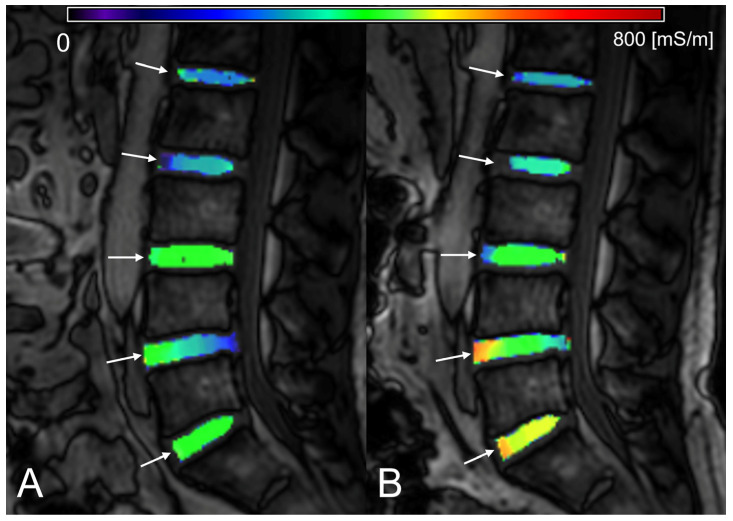
Diurnal variation in electrical conductivity (σ) of lumbar intervertebral discs (IVDs). Segmented ROIs of the morning scan (**A**) and evening scan (**B**) are shown overlaid on the magnitude images of electric properties tomography (EPT). The white arrows indicate IVDs. The look-up table represents σ value in mS/m. For most IVDs, σ of the evening scan is higher than the morning scan.

**Figure 5:**
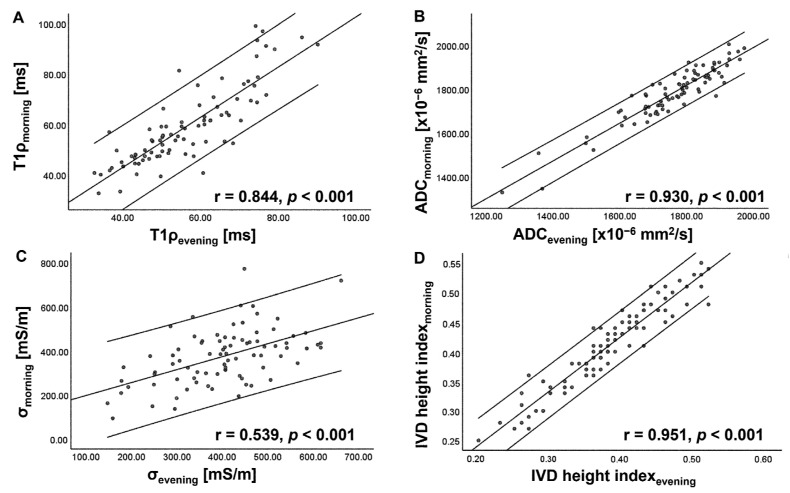
Scatterplots showing correlation of T1rho (T1ρ) (**A**), apparent diffusion coefficient (ADC) (**B**), electrical conductivity (σ) (**C**), and intervertebral disc (IVD) height index (**D**) between morning and evening scans. The center line indicates the mean, and the other two lines represent 95% confidence interval (CI). Significant moderate to very strong correlation is observed.

Table 1 and Figure S1 summarize the T1ρ, ADC, and σ values of the IVDs at each time point. The IVD height index and the breakdown of Pfirrmann grades are also given. In general, the T1ρ and ADC of IVDs decreased, whereas σ increased in the evening (*p* < 0.05) (Figure 2, Figure 3 and Figure 4). A significant decrease in the IVD height index in the evening was also observed (*p* < 0.001). For all quantitative MRI indices and IVD height index, the morning values correlated moderately to very strongly with the evening values (r = 0.539–0.951, *p* < 0.001) (Figure 5).

**Figure 6:**
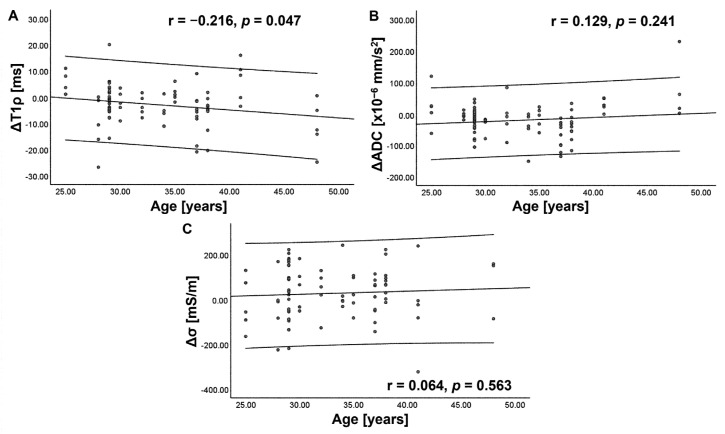
Scatterplots showing correlation of diurnal variation (Δ) in T1rho (T1ρ) (**A**), apparent diffusion coefficient (ADC) (**B**), and electrical conductivity (σ) (**C**) with age. The center line indicates the mean, and the other two lines represent 95% confidence interval (CI). A weak negative correlation is observed between ΔT1ρ and age.

**Figure 7:**
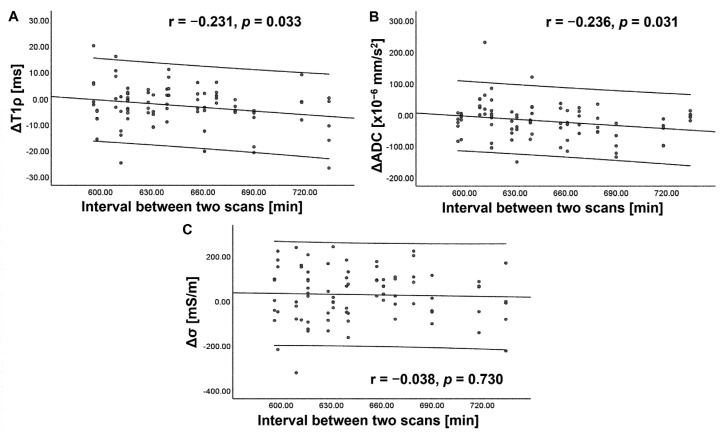
Scatterplots showing correlation of diurnal variation (Δ) in T1rho (T1ρ) (**A**), apparent diffusion coefficient (ADC) (**B**), and electrical conductivity (σ) (**C**) with scan interval. The center line indicates the mean, and the other two lines represent 95% confidence interval (CI). A weak negative correlation is observed between ΔT1ρ or ΔADC and scan interval.

The ΔT1ρ showed a weak negative correlation with age (r = −0.216, *p* = 0.047) and the scan interval (r = −0.231, *p* = 0.033) (Figure 6). ΔADC showed a weak negative correlation with the scan interval (r = −0.236, *p* = 0.031) (Figure 7). However, these correlations did not survive correction for multiple comparisons. No significant correlation was observed between the Δ of each index and that of other indices, Pfirrmann grade, or BMI (*p* > 0.05). For any index, the degree of Δ did not vary significantly with the IVD level (*p* = 0.910–1.000 for ΔT1ρ, *p* = 0.463–1.000 for ΔADC, and *p* = 0.107–1.000 for Δσ).

## 4. Discussion

This prospective study reports diurnal variation in the T1ρ, ADC, and σ of lumbar IVDs, in addition to the commonly known diurnal variation in IVD height index [13]. Diurnal variation exists in all three quantitative MRI indices, which implies that this variation needs to be accounted for when making a clinical decision based on these indices. With the increasing trend of incorporating quantitative MRI indices for diagnosing disease or assessing disease progression or treatment responsiveness [22,23,24], we expect that knowledge of normative values and their stability over time will become essential.

A decrease in IVD height in the evenings is a common phenomenon: a reduction in spinal height of up to almost 2 cm or about 1% of total body height can occur throughout the day while recovering during the night [12,25]. This day–night difference is induced by a diurnal change in intradiscal pressure according to the variation in day and night activity [26]. Hydrostatic pressure increases in an upright posture, resulting in fluid expression from the IVD [12,26]. This study observed a 7.5% reduction in the mean IVD height index.

It is reported that about 20–25% of the IVD’s water content is expressed and re-imbibed during each diurnal cycle [26]. This magnitude of change is sufficient to cause a change in ADC, which is highly sensitive to the diffusion of water molecules in and out of the cells [27]. In this study, ADC decreased significantly in the evening, amounting to a mean difference of 2.5% between the two time points. This result aligns with a previous study that reported an ADC decrease of 1.6% in NP and 5.2% in AF in the evenings [14]. Here, ADC decrease is thought to indicate fluid expression from IVDs.

T1ρ relaxation time, also known as the spin–lattice relaxation time in the rotating frame, is a time constant for transverse magnetization decay in a very weak B1 field strength produced by a spin-lock RF pulse [7]. Interaction between motion-restricted water molecules and their macromolecular environment seems to be the predominant contributing mechanism of T1ρ relaxation. Early biochemical changes in the macromolecular environment of IVD, such as proteoglycan loss, are reported as reflected in T1ρ values. Consistent with this, several studies have shown a decrease in T1ρ in degenerated IVD [7,28]. A negative relationship between T1ρ relaxation times and proteoglycan content in the NP has also been shown [29]. A few studies suggest the superiority of T1ρ to T2 and ADC in detecting IVD degeneration [30]. However, information about the diurnal variation in T1ρ has been lacking. This study observed a significant shortening in T1ρ relaxation time in the evenings, suggesting higher proteoglycan concentration within IVDs. The relative increase in proteoglycan concentration due to fluid expression from IVDs is thought to be attributable to the observed diurnal variation.

In contrast to other means of determining σ, σ estimation by EPT neither requires the injection of external currents nor the acquisition of different patient orientations. The potential usefulness of σ measured by EPT has been reported in brain and breast tumors and hepatic fibrosis [19,23,31]. In recent studies, the sensitivity of this technique to detect an exercise-induced change in the σ of muscles and the repeatability of the measurements have been documented [32]. To our knowledge, this is the first report on the σ values of lumbar IVDs. Like T1ρ and ADC, the diurnal variation in σ was also observed, with the evenings having higher σ values than the mornings. The exact underlying mechanism for this diurnal variation is yet to be discovered. However, from its high sensitivity to changes in sodium concentration, the observed difference may reflect a change in sodium ion concentration within IVDs. A previous study on sodium imaging of IVDs has shown a linear relationship between the sodium concentration and proteoglycan of IVDs [9]. In IVDs, proteoglycan molecules aggregate to form large proteoglycan aggrecans with around 100 glycosaminoglycan side chains. These side chains are extensively sulfated and carboxylated, which makes the whole molecule highly negatively charged at physiological pH. This negative charge attracts cations (primarily sodium ions). A relative increase in proteoglycan in the evenings may thus increase the local sodium ion concentration, thereby causing an increase in σ. It has to be kept in mind that the absolute σ value of IVD will be higher than the values reported in this study due to the mentioned specific type of simplifications in the EPT reconstruction applied. This specific type consists of reducing the number of spatial derivative directions from three to one. Assuming that each derivative direction contributes in first order equally to σ, an absolute value of σ three times higher than the observed values can be estimated. However, it is not expected that this missing factor impacts the observed diurnal variation in σ found in this study.

This study indicated a few correlations of the diurnal variation in the quantitative MRI indices with age and scan interval. The correlation of ΔT1ρ with age is thought to reflect the age-dependent diminution in intradiscal proteoglycan concentration, the finding of which is consistent with previous studies [3,33]. The correlation of ΔT1ρ or ΔADC with the scan interval was rather weak. In this study, the interval varied from 9.93 to 12.23 h. Longer axial loading may further accentuate the diurnal variation. The lack of a significant correlation of Δ among the three indices indicates that these indices are independent of one another. Their combination may better reflect the ongoing biochemical changes within IVD than either of them alone. A correlation of ΔADC with ΔIVD height index was expected [14]. However, only a trend toward correlation was observed (r = 0.214, *p* = 0.050). Failure to achieve a significant correlation may be due to a limitation in sample size. The lack of correlation of these changes to Pfirrmann grade or IVD level suggests that the degree of IVD degeneration or the IVD level do not affect diurnal variation. The lack of correlation to Pfirrmann grade can also be due to the inclusion of mostly Pfirrmann Grade III IVDs in the cohort. Finally, BMI has been reported to affect diurnal variation, which was not reproducible in this study [15]. In this study, 8.2%, 82.4%, and 9.4% of IVDs were classified as Pfirrmann Grade II, III, and IV, respectively. IVDs classified as Pfirrmann Grade I or V were lacking. We assume that this distribution is representative of the general population. Asymptomatic IVD degeneration is a common MRI observation. A previous report evaluating the prevalence of IVD degeneration in 1043 individuals between 18 and 55 years of age observed lumbar IVD degeneration in 40% of individuals under 30 [34]. Another study has shown a 56% prevalence of lumbar IVD degeneration in individuals between 15 and 30 years of age with no history of low back pain [35]. A study has suggested considering only Grade IV and Grade V as degenerative [36].

Several limitations need to be addressed. First, all measurements were performed using the mid-sagittal imaging plane. This choice was made to limit partial volume artifacts. The inclusion of all slices or volumetric measurements may be ideal. Second, our ROIs included both AF and NP. Separate measurements may enhance the understanding of dynamic changes occurring in the IVDs. However, separating AF and NP is difficult, especially in degenerated discs [4]. ROIs that include the whole IVD may be preferable in clinical settings. Third, although a weak correlation was observed between ΔT1ρ or ΔADC and age or scan interval, these findings did not hold after a correction for multiple comparisons. Further studies with larger sample sizes are necessary to prove these exploratory observations. Fourth, a simplified method was used in the reconstruction of σ maps. As mentioned earlier, the actual σ values of IVD will be larger than those reported in this study.

## 5. Conclusions

This study reports the diurnal variations in the T1ρ, ADC, and σ of lumbar IVDs in sedentary workers. There exists a diurnal variation in these quantitative MRI indices, reflective of daily physiological events. Knowledge of this variation will be important in interpreting these indices for clinical decisions. The results of this study may serve as the reference values for lumbar IVDs. Finally, this study proposed a method to noninvasively assess the σ of IVD.

## Figures and Tables

**Table 1:** T1rho (T1ρ), apparent diffusion coefficient (ADC), and electrical conductivity (σ) values of each lumbar intervertebral disc (IVD) at each time point. The intervertebral disc (IVD) height index and the breakdown of Pfirrmann grade are also given. All data except Pfirrmann grade are given in mean ± standard deviation (range). The *p*-values indicate statistical significance for comparisons of the indices of all lumbar IVDs or each lumbar IVD between the two time points. N indicates sample size. L1/2 refers to the IVD between the first and second lumbar vertebrae, L2/3 that between the second and third vertebrae, and so on. L5/S1 refers to the IVD between the fifth lumbar vertebra and sacrum.

		All IVDs	Individual IVD				
			L1/L2	L2/L3	L3/L4	L4/L5	L5/S1
**IVD height index**	**Morning**	0.410 ± 0.071	0.341 ± 0.049	0.403 ± 0.062	0.449 ± 0.044	0.461 ± 0.059	0.410 ± 0.071
		(0.25 − 0.55)	(0.28 − 0.47)	(0.27 − 0.54)	(0.37 − 0.53)	(0.30 − 0.55)	(0.25 − 0.51)
	**Evening**	0.382 ± 0.073	0.313 ± 0.057	0.374 ± 0.066	0.422 ± 0.054	0.428 ± 0.051	0.371 ± 0.074
		(0.20 − 0.52)	(0.23 − 0.45)	(0.25 − 0.52)	(0.36 − 0.52)	(0.29 − 0.51)	(0.20 − 0.51)
	**N**	85	17	17	17	17	17
	***p***	<0.001	<0.001	<0.006	<0.010	<0.001	0.001
							
**T1** **ρ** **[ms]**	**Morning**	59.069 ± 15.308	51.426 ± 8.338	61.095 ± 13.444	70.199 ± 16.177	63.409 ± 18.038	49.215 ± 8.467
		(32.6 − 98.98)	(40.0 − 70.05)	(32.66 − 87.01)	(48.0 − 98.98)	(39.3 − 96.88)	(33.3 − 70.78)
	**Evening**	56.292 ± 12.991	49.835 ± 10.490	59.103 ± 12.061	65.635 ± 11.930	58.293 ± 13.502	48.596 ± 9.657
		(32.7 − 90.19)	(32.7 − 69.47)	(33.8 − 74.57)	(43.3 − 86.13)	(36.7 − 90.19)	(36.57 − 70.42)
	**N**	85	17	17	17	17	17
	***p***	0.002	0.196	0.207	0.065	0.055	0.779
							
**ADC [×10^−6^ mm^2^/s]**	**Morning**	1784.633 ±124.527	1744.867 ±84.098	1810.641 ±116.277	1849.257 ±89.464	1776.74 1 ± 168.954	1738.196 ±127.040
		(1328.54 − 2005.07)	(1633.64 − 1909.40)	(1507.33 − 2005.07)	(1689.26 − 1986.82)	(1328.54 − 1922.59)	(1345.55 − 1872.46)
	**Evening**	1761.456 ±134.130	1728.474 ±63.042	1769.122 ±142.371	1824.274 ±103.646	1751.606 ±183.742	1733.224 ±142.604
		(1247.46 − 1972.04)	(1605.50 − 1849.15)	(1356.50 − 1926.90)	(1602.80 − 1972.04)	(1247.46 − 1897.12)	(1376.50 − 1912.06)
	**N**	84	17	17	17	16	17
	***p***	<0.001	0.101	0.002	0.025	0.056	0.741
							
**σ [mS/m]**	**Morning**	374.696 ±123.957	333.879 ±155.562	365.987 ±93.528	440.309 ±98.890	375.274 ±96.922	361.091 ±149.300
		(94.83 − 770.99)	(137.39 − 770.99)	(209.03 − 568.22)	(295.05 − 605.21)	(186.04 − 520.26)	(94.83 − 717.46)
	**Evening**	401.607 ±113.978	348.211 ±81.711	396.742 ±108.578	420.614 ±99.149	440.663 ±98.776	403.005 ±160.339
		(108.88 − 777.75)	(241.14 − 492.45)	(177.25 − 556.25)	(182.58 − 609.89)	(299.25 − 585.64)	(147.30 − 661.28)
	**N**	83	17	17	16	17	16
	***p***	0.035	0.554	0.236	0.492	0.010	0.134
							
**No. of IVDs with Pfirrmann grade**	**Grade II**	7	2	2	2	1	0
	**Grade III**	70	14	14	15	12	15
	**Grade IV**	8	1	1	0	4	2

Note: The *p*-values of *t*-tests for each IVD level are shown in gray.

## Data Availability

The data are not publicly available to ensure data security and for ethical restrictions but are available from the corresponding author on reasonable request.

## Supplementary Materials

The following are available online at https://www.mdpi.com/article/10.3390/tomography9030084/s1, Figure S1: Intervertebral disc (IVD) height index (A), T1rho (T1ρ) (B), apparent diffusion coefficient (ADC) (C), and electrical conductivity (σ) (D) values of lumbar intervertebral discs (IVDs) at each time point and Pfirrmann Grade.
