# Supplementary material for: Quantitative Assessment of Intervertebral Disc Composition by MRI: Sensitivity to Diurnal Variation
Source: Tomography. 2023 May 16;9(3):1029–40. doi: 10.3390/tomography9030084 (PMC10204382; doi:10.3390/tomography9030084)
Supplement: Supplementary file 1 [file tomography-09-00084-s001.zip › tomography-2384149-supplementary.pdf]

Supplementary Material

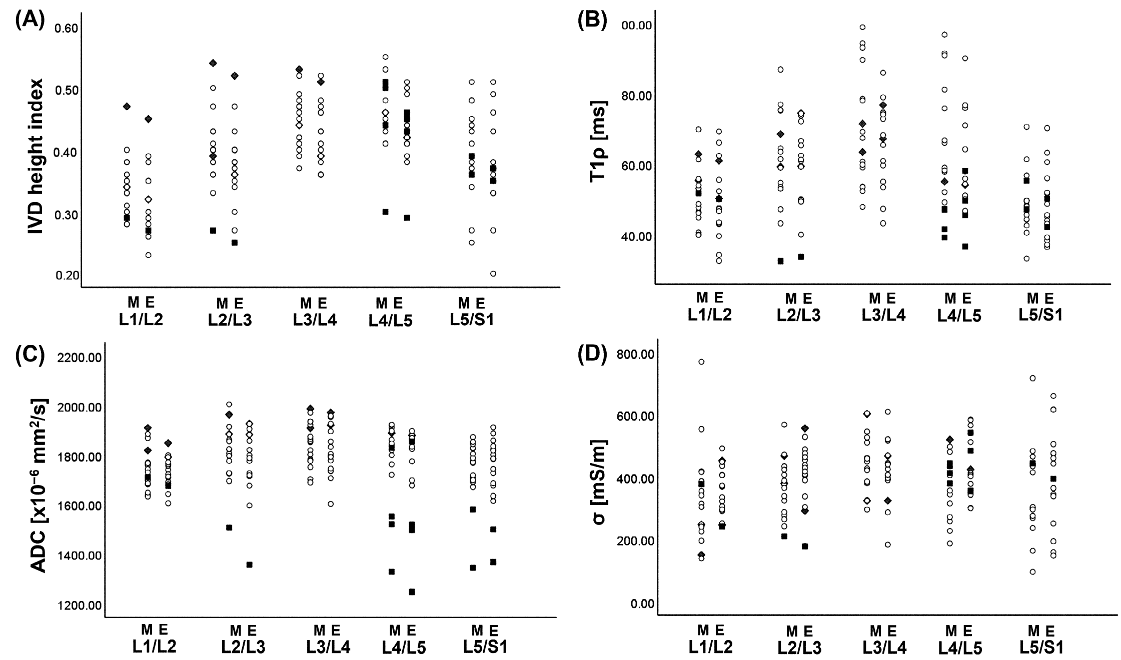

Figure S1. Intervertebral disc (IVD) height index (**A**), T1rho (T1ρ) (**B**), apparent diffusion coefficient (ADC) (**C**), and electrical conductivity (σ) (**D**) values of lumbar intervertebral discs (IVDs) at each time point and Pfirrmann Grade. ◆, ○, and ■ indicate Grade II, Grade III, and Grade IV, respectively. L1/2 refers to the IVD between the first and second lumbar vertebrae, L2/3 that between the second and third vertebrae, and so on. L5/S1 refers to the IVD between the fifth lumbar vertebra and sacrum. M and E refer to morning and evening scans, respectively.
